# GeneTalk: an expert exchange platform for assessing rare sequence variants in personal genomes

**DOI:** 10.1093/bioinformatics/bts462

**Published:** 2012-07-23

**Authors:** Tom Kamphans, Peter M. Krawitz

**Affiliations:** ^1^GeneTalk, Finckensteinallee 84, 12205 Berlin and ^2^Department of Medical and Human Genetics, Charité, Univfersitätsmedizin Berlin, Augustenburger Platz 1, 13353 Berlin, Germany

## Abstract

**Summary**: Next-generation sequencing has become a powerful tool in personalized medicine. Exomes or even whole genomes of patients suffering from rare diseases are screened for sequence variants. After filtering out common polymorphisms, the assessment and interpretation of detected personal variants in the clinical context is an often time-consuming effort. We have developed GeneTalk, a web-based platform that serves as an expert exchange network for the assessment of personal and potentially disease-relevant sequence variants. GeneTalk assists a clinical geneticist who is searching for information about specific sequence variants and connects this user to other users with expertise for the same sequence variant.

**Availability**: GeneTalk is available at www.gene-talk.de. Users can login without registering in a demo account.

**Contact**: peter.krawitz@gene-talk.de

## 1 INTRODUCTION

Exome sequencing has become an invaluably powerful tool in the identification of disease causing variants (Bamshad *et al.*, 2011; Robinson *et al.*, 2011) and the first patients are now already treated based on sequence variant information of their exomes (Worthey *et al.*, 2011). To date, the primary bottleneck in such clinical personal genome cases is not anymore data generation but data analysis. Clinical geneticists therefore require efficient tools for filtering and interpreting the clinically meaningful sequence variants.

Currently, the most potent filters for reducing the set of novel and potentially causal mutations in rare diseases are based on variation data from population scale sequencing efforts such as the 1000 genomes project (www.1000genomes.org). Only very few variants will be medically relevant, perhaps—in case of a monogenic disorder—just one. Bioinformatics tools such as ANNOVAR (Wang *et al.*, 2010) and MutationTaster (Schwarz *et al.*, 2010) may then be used for comprehensive annotations and predictions about the expected pathogenicity of a variant. However, such classifiers have high false-positive and -negative error rates. They may therefore serve only for prioritization but cannot replace the assessment of human experts.

## APPROACH

In a genetic disease of unknown cause, the association with a new disease gene may be shown either by a functional assessment of the detected variants or by statistical evidence. For a functional assessment, expert knowledge about a suitable test essay or genetically modified model organisms is required. For statistical evidence, a sufficient number of patients of the same disease group with mutations in the same gene are required. However, usually a clinical geneticist analyzing a patient’s exome does not have access to many similar cases because the disease is so rare. Furthermore, she may not be skilled to do the functional assessment immediately and happened to identify a new gene of interest after filtering the patient’s variants. Hence, such a geneticist is interested in finding other individuals with mutations in the same gene or scientists that are performing basic research on this gene. Web-based expert networks proved to be efficient tools for knowledge management in various scientific fields. There are knowledge bases for disease, gene and protein-centered information (www.ncbi.nlm.nih.gov/omim, www.geneontology.org, www.wikiproteins.org). However, there is no platform that allows the scientific exchange of experts about specific variants detected in next-generation sequencing (NGS) experiments. GeneTalk aims at providing such a web-based platform that enables to improve expert annotations on human genetic variants in a community approach.

## APPLICATION

**Fig. 1:**
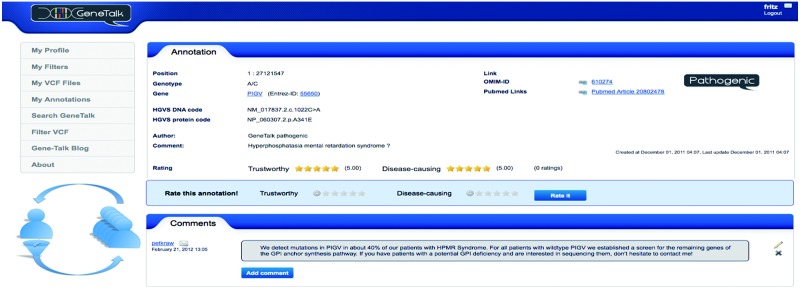
GeneTalk, a communication platform for sequence variants. A user filters sequence variants down to a small set of potentially disease relevant mutations. She then searches for detailed information annotated by the GeneTalk community for these variants. In GeneTalk, users may annotate and comment genetic variants. Annotations and comments may link to the relevant literature or discuss experimental and clinical findings. Based on this locus-specific information, GeneTalk users may rate the trustworthiness of an annotation and the potential of a mutation to be disease causing. This screenshot is taken from fritz’ account who is looking at the annotation of a mutation in the gene PIGV. The GeneTalk community finds this annotation trustworthy and rates the described mutation as highly likely to cause a syndrome called hyperphosphatasia with mental retardation. The user petkraw left a comment for this variant. He seems to have some expertise in this disease and might be an interesting person to contact for fritz

GeneTalk is an exchange platform that allows users to look for variant-specific information and makes human expertise searchable (Fig. 1). Any sequence variant with respect to the human reference genome, based on the GRCh37 assembly, is annotatable. The user decides to whom an annotation is visible. One user may link to scientific articles that are relevant in context with a certain variant or that even provide evidence that a mutation is disease causing. A second user might comment on this annotation to express her concern because she views the detected variant as an technical artefact. A third user might state that she has seen patients with this genotype and is not sure about the statistical significance of the association with the phenotype. All annotations and comments of GeneTalk users about a certain genomic position can be read like a locus-specific conversation thread. The trustworthiness of annotations can be rated by users as well as the likelihood of a mutation to be disease causing (Fig. 1). If there is consensus in the GeneTalk community that a certain mutation is pathogenic and its annotation is trustworthy, this mutation is added to the annotation track ‘pathogenic’. This annotation track is thus curated in a collaborative effort of all GeneTalk users.

GeneTalk also assists users in filtering genetic variants from NGS projects. A user that has a patient’s informed consent to analyze the clinical data may upload sequence variants to GeneTalk in variant call format (VCF) (Danecek *et al.*, 2011), version 4.0 and above. In order to reduce the initial VCF to a set of potentially disease relevant mutations, the user can apply certain filter settings first: the list of variants could be restricted to, e.g. only nonsynonymous, homozygous variants with the functional and inheritance filter. Common variants can be filtered out by a genotype frequency filter that is based on high-quality NGS data sets from HapMap, the 1000 genomes project and the 5000 exomes project. If a linkage analysis has been performed, a genomic interval may be set to limit the search space or gene panels may be applied as *in silico* filters to restrict the analysis to certain molecular pathways. In a rare recessive monogenic disease, the mode of inheritance and the genotype frequency filter that is set to 1/1000 usually reduce the number of candidate mutations down to a few hundreds in a patient’s exome. These variants may then be further analyzed for ‘disease-causing’ annotations. If the pathogenic mutation of this case has not yet been described in the literature and no ‘pathogenic’ annotation exists, the user can look for annotations that discuss patients with similar phenotypes or basic research scientists that talk about unpublished experimental data for this gene. Such an annotation can serve as a conversation starter and the users can simply contact the author by clicking on the envelope symbol (Fig. 1). Currently, the annotation database contains over 32 000 clinically relevant entries from dBSNP. A video tutorial on www.gene-talk.de illustrates how an exome dataset may be analyzed in GeneTalk: In a few easy steps, the variant data of a simulated patient with hyperphosphatasia with mental retardation syndrome are filtered down to the disease-causing mutation.

## CONCLUSIONS

GeneTalk provides an intuitive web-based interface for geneticists that analyze human sequence variants. GeneTalk is a platform for efficient knowledge management of genetic variants and simplifies the scientific discussion and interpretation especially of rare mutations.
